# Long COVID and Recovery Among US Adults

**DOI:** 10.1001/jamanetworkopen.2026.0374

**Published:** 2026-03-02

**Authors:** Rishi M. Shah, Kavya M. Shah, Ji Chen, Mitsuaki Sawano, Bornali Bhattacharjee, Harlan M. Krumholz

**Affiliations:** 1 Center for Outcomes Research and Evaluation, Yale New Haven Hospital, New Haven, Connecticut; 2 Yale College, New Haven, Connecticut; 3 Harvard Medical School, Boston, Massachusetts; 4 Section of Cardiovascular Medicine, Department of Internal Medicine, Yale School of Medicine, New Haven, Connecticut; 5 Department of Immunobiology, Yale School of Medicine, New Haven, Connecticut; 6 Center for Infection and Immunity, Yale School of Medicine, New Haven, Connecticut; 7 Department of Health Policy and Management, Yale School of Public Health, New Haven, Connecticut

## Abstract

This cross-sectional study examines the prevalence and recovery patterns of long COVID among US adults using data from the National Health Interview Survey.

## Introduction

Long COVID (LC) remains a public health challenge, with millions of US adults continuing to experience its debilitating effects. Although studies have identified characteristics linked to LC and recovery from LC,^1,2^ questions remain about its scope and trajectory. Using nationally representative data, we estimate LC prevalence and recovery and assess how associated factors have evolved.

## Methods

We used data from the 2022 to 2024 National Health Interview Survey (NHIS), an annual cross-sectional survey representative of the US noninstitutionalized civilian population.^3^ We used NHIS-provided sampling weights, strata, and primary sampling units to yield nationally representative estimates.

Our sample consisted of adults 18 years or older who self-reported either a positive COVID-19 test or a physician’s diagnosis of COVID-19 and had complete data on LC and recovery status. LC was defined as an affirmative response to the question, “Did you have any symptoms lasting 3 months or longer that you did not have prior to having coronavirus or COVID-19?” Recovery from LC was defined as an affirmative response to the previous question and a negative response to the question, “Do you have symptoms now?” (asked as a follow-up to those ever-reporting LC). We compared sociodemographic characteristics between adults with and without LC, and between those who had and had not recovered, using Rao-Scott χ^2^ tests (2-sided *P* < .05 indicating significance). Multivariable logistic regression was used to identify factors associated with LC and recovery, with potential covariates chosen based on prior knowledge of sociodemographic factors associated with COVID-19 infection.^1,2^ Multiplicity adjustment was not performed, given the study’s descriptive nature. Analyses were conducted using R, version 4.3.1 (R Foundation) with the survey package. This study was exempt from review by the Yale University Institutional Review Board because data are publicly available and deidentified. Analyses followed STROBE guidelines.

## Results

Our sample included 9022 adults (weighted mean [SE] age, 44.4 [0.21] years; weighted 52.7% female) in 2022, 15 354 (45.7 [0.19] years; 53.0% female) in 2023, and 18 691 (46.3 [0.18] years; 53.6% female) in 2024. The weighted proportion of US adults reporting prior COVID-19 infection rose from 39.6% (95% CI, 38.8%-40.3%) in 2022 to 60.4% (95% CI, 59.7%-61.1%) in 2024. Among those infected, the weighted prevalence of LC since the onset of the pandemic declined from 19.7% (95% CI, 18.7%-20.7%) to 13.7% (95% CI, 13.1%-14.4%), while weighted LC recovery rates rose from 51.2% (95% CI, 48.4%-53.9%) to 59.7% (95% CI, 57.1%-62.2%).

Female sex, age 35 to 64 years, and low household income were consistently associated with higher odds of LC, while non-Hispanic Asian adults had lower odds (Table 1). Recovery from LC remained less likely among adults 35 years or older. No consistent differences by sex, race and ethnicity, education, urbanicity, employment status, household income, insurance status, usual source of care, or US region were observed (Table 2).

**Table 1.** Adjusted Odds Ratios (AORs) for Sociodemographic Characteristics of US Adults With and Without Long COVID (LC)^a^

Characteristic^b^	Year											
	2022				2023				2024			
	No LC, No. (%) (n = 7149)	LC, No. (%) (n = 1797)	*P* value^c^	AOR (95% CI)	No LC, No. (%) (n = 12 893)	LC, No. (%) (n = 2398)	*P* value^c^	AOR (95% CI)	No LC, No. (%) (n = 16 008)	LC, No. (%) (n = 2627)	*P* value^c^	AOR (95% CI)
Age group, y												
18-34	2053 (36)	416 (29)	<.001	1 [Reference]	3253 (33)	526 (28)	<.001	1 [Reference]	3804 (31)	578 (29)	.006	1 [Reference]
35-49	1985 (28)	525 (31)		1.60 (1.35-1.89)	3296 (27)	642 (29)		1.36 (1.16-1.59)	4044 (27)	699 (29)		1.19 (1.04-1.37)
50-64	1683 (22)	483 (27)		1.64 (1.38-1.95)	3110 (23)	675 (27)		1.48 (1.27-1.73)	3804 (23)	693 (25)		1.18 (1.02-1.37)
≥65	1417 (14)	370 (13)		1.15 (0.92-1.44)	3216 (17)	554 (16)		1.00 (0.84-1.19)	4336 (19)	651 (17)		0.91 (0.76-1.09)
Sex												
Male	3270 (49)	638 (37)	<.001	1 [Reference]	5902 (49)	839 (37)	<.001	1 [Reference]	7258 (48)	926 (38)	<.001	1 [Reference]
Female	3879 (51)	1159 (63)		1.53 (1.34-1.74)	6991 (51)	1559 (63)		1.49 (1.34-1.67)	8750 (52)	1701 (62)		1.46 (1.31-1.63)
Race and ethnicity												
Hispanic	1187 (19)	321 (21)	<.001	0.96 (0.79-1.17)	2060 (18)	419 (20)	<.001	0.98 (0.84-1.15)	2427 (18)	402 (18)	<.001	0.87 (0.74-1.01)
Non-Hispanic Asian	407 (5)	44 (2)		0.40 (0.28-0.58)	766 (6)	77 (3)		0.57 (0.43-0.76)	1004 (6)	86 (4)		0.59 (0.44-0.77)
Non-Hispanic Black	663 (10)	162 (9)		0.80 (0.64-1.00)	1130 (10)	208 (9)		0.86 (0.70-1.05)	1252 (9)	220 (10)		0.95 (0.78-1.16)
Non-Hispanic White	4703 (63)	1209 (64)		1 [Reference]	8625 (64)	1622 (64)		1 [Reference]	10 874 (64)	1817 (65)		1 [Reference]
Other^d^	189 (3)	61 (4)		1.27 (0.89-1.81)	312 (2)	72 (3)		1.07 (0.78-1.46)	451 (3)	102 (4)		1.09 (0.83-1.43)
Education												
College degree	3992 (50)	913 (47)	.20	1 [Reference]	7271 (51)	1227 (47)	.007	1 [Reference]	9175 (53)	1382 (49)	.03	1 [Reference]
HS diploma	2671 (42)	748 (43)		0.99 (0.87-1.14)	4702 (40)	986 (44)		1.04 (0.91-1.19)	5835 (40)	1054 (43)		1.02 (0.90-1.15)
<HS	461 (8)	128 (10)		0.98 (0.73-1.31)	869 (9)	175 (9)		0.81 (0.63-1.03)	936 (8)	181 (9)		1.02 (0.81-1.29)
Urbanicity^e^												
Metropolitan	6112 (87)	1481 (85)	.02	1 [Reference]	11 089 (87)	1969 (84)	<.001	1 [Reference]	12 807 (87)	1933 (85)	<.001	1 [Reference]
Nonmetropolitan	1037 (13)	316 (15)		1.12 (0.94-1.33)	1804 (13)	429 (16)		1.18 (1.01-1.38)	3201 (13)	694 (17)		1.23 (1.06-1.44)
Employment status												
Employed	4899 (75)	1152 (71)	.002	1 [Reference]	8339 (71)	1443 (66)	<.001	1 [Reference]	10 118 (69)	1629 (68)	.39	1 [Reference]
Unemployed	1992 (25)	579 (29)		1.07 (0.91-1.25)	4073 (29)	876 (34)		1.13 (0.98-1.30)	5374 (31)	930 (32)		0.99 (0.86-1.14)
Household income												
≥400% FPL	3513 (48)	712 (39)	<.001	1 [Reference]	6233 (48)	908 (38)	<.001	1 [Reference]	7847 (49)	1005 (39)	<.001	1 [Reference]
200%-399% FPL	2034 (29)	565 (33)		1.41 (1.21-1.64)	3711 (29)	736 (31)		1.30 (1.14-1.48)	4626 (29)	849 (33)		1.45 (1.27-1.66)
<200% FPL	1602 (23)	520 (28)		1.44 (1.19-1.74)	2949 (23)	754 (31)		1.49 (1.27-1.74)	3535 (22)	773 (28)		1.56 (1.32-1.83)
Insurance status												
Private	4941 (70)	1145 (65)	<.001	1 [Reference]	8826 (70)	1477 (62)	<.001	1 [Reference]	10 927 (70)	1686 (67)	.10	1 [Reference]
Public	1677 (21)	513 (26)		1.19 (1.00-1.41)	3247 (23)	742 (29)		1.24 (1.08-1.42)	4070 (23)	754 (25)		1.00 (0.87-1.16)
Uninsured	515 (9)	137 (9)		1.07 (0.83-1.38)	781 (7)	172 (9)		1.23 (0.98-1.54)	978 (8)	182 (8)		1.01 (0.82-1.25)
Usual source of care												
Yes	6412 (89)	1636 (91)	.06	1 [Reference]	11 595 (89)	2149 (89)	.62	1 [Reference]	14 630 (90)	2413 (91)	.39	1 [Reference]
No	726 (11)	155 (9.0)		0.91 (0.72-1.15)	1263 (11)	243 (11)		1.06 (0.87-1.29)	1345 (10)	209 (9.0)		0.93 (0.76-1.14)
US region												
Northeast	1263 (19)	277 (17)	.09	1 [Reference]	2193 (19)	317 (15)	.002	1 [Reference]	2743 (19)	366 (15)	.005	1 [Reference]
Midwest	1599 (21)	433 (22)		1.02 (0.82-1.26)	2741 (20)	527 (21)		1.24 (1.03-1.50)	3545 (20)	633 (22)		1.27 (1.05-1.53)
South	2642 (38)	643 (36)		0.91 (0.74-1.13)	4570 (36)	934 (39)		1.31 (1.11-1.55)	5566 (37)	930 (38)		1.20 (1.01-1.42)
West	1645 (22)	444 (25)		1.17 (0.93-1.46)	3389 (25)	620 (25)		1.31 (1.10-1.57)	4154 (25)	698 (26)		1.33 (1.11-1.59)

Abbreviations: FPL, federal poverty level; HS, high school.

^a^ Individuals who ever reported COVID-19 but had an unknown or missing response to the LC status question were excluded from the analytic sample (2022: 76 of 9022 [0.8%]; 2023: 63 of 15 354 [0.4%]; 2024: 56 of 18 691 [0.3%]).

^b^ Respondent counts are unweighted; percentage estimates and 95% CIs are weighted and rounded and therefore may not always sum to 100%.

^c^ Pearson χ^2^ tests with second-order Rao-Scott correction. The multivariable logistic regression model adjusted for only the covariates described in the table.

^d^ Other includes non-Hispanic American Indian or Alaska Native only, non-Hispanic American Indian or Alaska Native in combination with another race, and non-Hispanic respondents reporting other single or multiple races.

^e^ Metropolitan includes large central, large fringe, medium, and small metropolitan counties; nonmetropolitan includes micropolitan and noncore counties with populations less than 50 000 residents.

**Table 2.** Adjusted Odds Ratios (AORs) for Sociodemographic Characteristics of Adults Currently With and Recovered from Long COVID (LC)^a^

Characteristic^b^	Year											
	2022				2023				2024			
	Not recovered, No. (%) (n = 919)	Recovered, No. (%) (n = 872)	*P* value^c^	AOR (95% CI)	Not recovered, No. (%) (n = 1063)	Recovered, No. (%) (n = 1319)	*P* value^c^	AOR (95% CI)	Not recovered, No. (%) (n = 1106)	Recovered, No. (%) (n = 1501)	*P* value^c^	AOR (95% CI)
Age group, y												
18-34	167 (23)	247 (33)	<.001	1 [Reference]	184 (23)	339 (32)	<.001	1 [Reference]	175 (22)	399 (34)	<.001	1 [Reference]
35-49	281 (34)	243 (29)		0.57 (0.42-0.78)	256 (28)	385 (30)		0.71 (0.52-0.97)	258 (28)	436 (30)		0.71 (0.54-0.94)
50-64	257 (28)	223 (26)		0.65 (0.48-0.90)	318 (29)	353 (25)		0.60 (0.46-0.79)	323 (29)	365 (22)		0.50 (0.38-0.67)
≥65	211 (15)	158 (12)		0.68 (0.46-1.00)	305 (20)	241 (13)		0.65 (0.48-0.89)	348 (21)	298 (14)		0.45 (0.32-0.63)
Sex												
Male	295 (33)	341 (40)	.001	1 [Reference]	347 (34)	486 (40)	.02	1 [Reference]	380 (35)	538 (40)	.07	1 [Reference]
Female	624 (67)	531 (60)		0.77 (0.60-0.97)	716 (66)	833 (60)		0.81 (0.65-1.00)	726 (65)	963 (60)		0.86 (0.70-1.05)
Race and ethnicity												
Hispanic	133 (17)	187 (24)	.02	1.47 (1.08-1.99)	168 (19)	250 (21)	.61	1.29 (0.95-1.73)	137 (16)	264 (20)	.19	1.23 (0.90-1.67)
Non-Hispanic Asian	21 (2)	23 (3)		1.47 (0.73-2.93)	28 (3)	47 (3)		0.99 (0.54-1.83)	26 (3)	60 (4)		1.59 (0.88-2.87)
Non-Hispanic Black	75 (8)	86 (10)		1.66 (1.11-2.50)	90 (9)	118 (10)		1.31 (0.90-1.89)	97 (10)	122 (9)		0.92 (0.62-1.38)
Non-Hispanic White	661 (69)	544 (59)		1 [Reference]	745 (67)	865 (63)		1 [Reference]	803 (68)	996 (63)		1 [Reference]
Other^d^	29 (4)	32 (5)		1.45 (0.66-3.17)	32 (3)	39 (3)		1.08 (0.63-1.85)	43 (3)	59 (4)		1.11 (0.63-1.93)
Education												
College degree	457 (47)	456 (48)	.01	1 [Reference]	511 (43)	708 (49)	.01	1 [Reference]	565 (49)	805 (49)	.60	1 [Reference]
HS diploma	406 (46)	337 (41)		0.93 (0.72-1.20)	460 (46)	518 (44)		0.87 (0.70-1.08)	461 (43)	585 (42)		0.93 (0.75-1.16)
<HS	54 (7)	74 (12)		1.68 (0.99-2.85)	88 (11)	87 (7)		0.67 (0.43-1.04)	74 (8)	107 (9)		1.21 (0.76-1.91)
Urbanicity^e^												
Metropolitan	732 (83)	743 (86)	.18	1 [Reference]	855 (82)	1101 (85)	.08	1 [Reference]	775 (81)	1142 (85)	.03	1 [Reference]
Nonmetropolitan	187 (17)	129 (14)		0.91 (0.65-1.29)	208 (18)	218 (15)		0.89 (0.68-1.16)	331 (19)	359 (15)		0.85 (0.67-1.07)
Employment status												
Employed	566 (68)	582 (72)	.05	1 [Reference]	556 (57)	879 (72)	<.001	1 [Reference]	637 (65)	981 (71)	.006	1 [Reference]
Unemployed	317 (32)	260 (28)		0.89 (0.67-1.18)	483 (43)	388 (28)		0.63 (0.49-0.80)	446 (35)	475 (29)		0.99 (0.77-1.27)
Household income												
≥400% FPL	354 (39)	355 (39)	.83	1 [Reference]	373 (36)	531 (40)	.008	1 [Reference]	407 (39)	592 (39)	.99	1 [Reference]
200%-399% FPL	291 (32)	274 (34)		0.96 (0.73-1.27)	315 (29)	414 (32)		0.99 (0.78-1.26)	351 (33)	489 (34)		0.98 (0.77-1.25)
<200% FPL	274 (29)	243 (27)		1.00 (0.71-1.41)	375 (35)	374 (28)		0.86 (0.65-1.14)	348 (28)	420 (28)		0.95 (0.71-1.26)
Insurance status												
Private	582 (65)	558 (65)	.05	1 [Reference]	609 (59)	859 (64)	<.001	1 [Reference]	692 (66)	982 (68)	.08	1 [Reference]
Public	281 (28)	231 (24)		0.80 (0.61-1.06)	388 (34)	348 (26)		0.90 (0.71-1.16)	349 (27)	397 (23)		0.95 (0.73-1.25)
Uninsured	55 (7.4)	82 (11)		1.13 (0.71-1.81)	63 (6.8)	108 (10)		1.55 (1.00-2.41)	63 (7.2)	119 (9.3)		1.21 (0.78-1.88)
Usual source of care												
Yes	846 (92)	784 (89)	.19	1 [Reference]	963 (90)	1172 (88)	.30	1 [Reference]	1024 (92)	1370 (91)	.55	1 [Reference]
No	69 (8.3)	86 (11)		1.12 (0.73-1.72)	97 (10)	144 (12)		0.99 (0.68-1.42)	81 (8.5)	127 (9.3)		0.81 (0.56-1.18)
US region												
Northeast	130 (15)	143 (18)	.14	1 [Reference]	144 (15)	171 (15)	.90	1 [Reference]	144 (15)	221 (15)	.81	1 [Reference]
Midwest	226 (22)	206 (23)		0.90 (0.62-1.31)	233 (20)	288 (21)		1.05 (0.73-1.50)	281 (21)	348 (22)		1.10 (0.76-1.59)
South	351 (39)	291 (33)		0.66 (0.45-0.96)	421 (39)	509 (39)		0.99 (0.72-1.36)	393 (37)	532 (38)		1.09 (0.77-1.53)
West	212 (24)	232 (26)		0.82 (0.55-1.23)	265 (26)	351 (25)		0.89 (0.62-1.27)	288 (27)	400 (25)		0.94 (0.66-1.34)

Abbreviations: FPL, federal poverty level; HS, high school.

^a^ Individuals who ever reported having LC but had an unknown or missing response to current symptom status were excluded from the analytic sample.

^b^ Respondent counts are unweighted; percentage estimates and 95% CIs are weighted and rounded and therefore may not always sum to 100%.

^c^ Pearson χ^2^ tests with second-order Rao-Scott correction. The multivariable logistic regression model adjusted for only the covariates described in the table.

^d^ Other includes non-Hispanic American Indian or Alaska Native only, non-Hispanic American Indian or Alaska Native in combination with another race, and non-Hispanic respondents reporting other single or multiple races.

^e^ Metropolitan includes large central, large fringe, medium, and small metropolitan counties; nonmetropolitan includes micropolitan and noncore counties with populations less than 50 000 residents.

## Discussion

In 2024, 8.3% of US adults—an estimated 21.3 million—reported ever having LC, among whom nearly 6 in 10 reported recovery, consistent with RECOVER initiative^4^ findings showing similar LC prevalence in 2023 and 2024 and longitudinal Veterans Affairs data demonstrating declining LC prevalence.^5^ Yet many adults, particularly those 35 years or older, continue to experience lasting symptoms. With no LC treatment demonstrating clear efficacy, greater investment in understanding biological mechanisms, including immunotypic differences between those who recover and those who do not, may provide insights into pathways of persistence and potential targets for intervention.^6^

This study has limitations. Self-reported LC and recovery may be misclassified, potentially biasing associations with sociodemographic factors; individuals with intermittent symptoms may be misclassified as recovered, inflating recovery estimates; the repeated cross-sectional design across survey years may reflect population-level prevalence rather than individual recovery trajectories; timing of acute COVID-19 infection and recovery was unavailable, precluding inclusion of time-dependent covariates such as COVID-19 vaccination status; and the narrow definition of LC, assessed at any time since the pandemic’s start, may affect prevalence estimates. Nevertheless, self-report remains the only way to assess LC in population data.

## Notes

Supplement.
Data Sharing Statement
