# Supplementary material for: Long COVID and Recovery Among US Adults
Source: JAMA Netw Open. 2026 Mar 2;9(3):e260374. doi: 10.1001/jamanetworkopen.2026.0374 (PMC12954541; doi:10.1001/jamanetworkopen.2026.0374)
Supplement: Supplement. — Data Sharing Statement [file jamanetwopen-e260374-s001.pdf]

Data Sharing Statement

Shah. Long COVID and Recovery Among US Adults. *JAMA Netw Open*. Published March 02, 2026. doi:10.1001/jamanetworkopen.2026.0374

Data
Data available: Yes
Data types: Deidentified participant data
How to access data: The data used in this study are publicly available and can be accessed at https://www.cdc.gov/nchs/nhis/. Code to reproduce the analyses in this study is available at reasonable request to the corresponding author.
When available: With publication

Supporting Documents
Document types: None

Additional Information
Who can access the data: Data are publicly available at https://www.cdc.gov/nchs/nhis/.
Types of analyses: For any purpose in accordance with the data use guidelines set forth by the CDC (see: https://www.cdc.gov/nchs/nhis/).
Mechanisms of data availability: Data are publicly available at https://www.cdc.gov/nchs/nhis/.
